# Preliminary Evidence for the Clinical Utility of Tactile Somatosensory Assessments of Sport-Related mTBI

**DOI:** 10.1186/s40798-021-00340-8

**Published:** 2021-08-09

**Authors:** Joshua P. McGeown, Patria A. Hume, Stephen Kara, Doug King, Alice Theadom

**Affiliations:** 1 Sports Performance Research Institute New Zealand (SPRINZ), Faculty of Health and Environmental Science, Auckland University of Technology, Private Bag 92006, Auckland, 1142 New Zealand; 2 Traumatic Brain Injury Network, Auckland University of Technology, Auckland, New Zealand; 3 Axis Sports Medicine Clinic, Auckland, New Zealand; 4 School of Science and Technology, University of New England, Armidale, NSW Australia

**Keywords:** Somatosensory, mTBI, Modelling, Prediction, Diagnosis, Recovery, Brain Gauge

## Abstract

**Objectives:**

To evaluate the clinical utility of tactile somatosensory assessments to assist clinicians in diagnosing sport-related mild traumatic brain injury (SR-mTBI), classifying recovery trajectory based on performance at initial clinical assessment, and determining if neurophysiological recovery coincided with clinical recovery.

**Research Design:**

Prospective cohort study with normative controls.

**Methods:**

At admission (*n* = 79) and discharge (*n* = 45/79), SR-mTBI patients completed the SCAT-5 symptom scale, along with the following three components from the Cortical Metrics Brain Gauge somatosensory assessment (BG-SA): temporal order judgement (TOJ), TOJ with confounding condition (TOJc), and duration discrimination (DUR). To assist SR-mTBI diagnosis on admission, BG-SA performance was used in logistic regression to discriminate cases belonging to the SR-mTBI sample or a healthy reference sample (pooled BG-SA data for healthy participants in previous studies). Decision trees evaluated how accurately BG-SA performance classified SR-mTBI recovery trajectories.

**Results:**

BG-SA TOJ, TOJc, and DUR poorly discriminated between cases belonging to the SR-mTBI sample or a healthy reference sample (0.54–0.70 AUC, 47.46–64.71 PPV, 48.48–61.11 NPV). The BG-SA evaluated did not accurately classify SR-mTBI recovery trajectories (> 14-day resolution 48%, ≤14–day resolution 54%, lost to referral/follow-up 45%). Mann-Whitney U tests revealed differences in BG-SA TOJc performance between SR-mTBI participants and the healthy reference sample at initial clinical assessment and at clinical recovery (*p* < 0.05).

**Conclusions:**

BG-SA TOJ, TOJc, and DUR appear to have limited clinical utility to assist clinicians with diagnosing SR-mTBI or predicting recovery trajectories under ecologically valid conditions. Neurophysiological abnormalities persisted beyond clinical recovery given abnormal BG-SA TOJc performance observed when SR-mTBI patients achieved clinical recovery.

## Key Points

- There is limited initial evidence for the use of BG-SA TOJ, TOJc, and DUR to assist diagnostic decision-making or to predict SR-mTBI recovery trajectory under ecologically valid conditions.
- BG-SA TOJc values did not return to the healthy reference sample levels at clinical recovery indicating incomplete neurophysiological recovery.
- Findings do not provide sufficient justification to recommend the allocation of time and resources to acquire BG-SA TOJ, TOJc, or DUR to assist clinical management of SR-mTBI patients at this time.

## Introduction

Mild traumatic brain injuries (mTBIs) sustained during sport or physical activity represent an estimated 20–25% of all traumatic brain injuries [1, 2]. mTBI is commonly described as an invisible injury because structural abnormalities are not detected post-mTBI using standard neuroimaging techniques [3, 4]. The invisible nature of mTBI requires that diagnosis be made via clinical examination where self-reported symptoms are one of the key indicators used by clinicians [5, 6]. Reliance on self-reported symptoms can be problematic because of the non-specific nature of mTBI symptoms and delayed symptom onset in some patients [5, 7–11]. In the case of sport-related mTBI (SR-mTBI), some athletes underreport when they have sustained an mTBI and/or minimise related symptomology [12–15]. Taken together, these limitations highlight the need for objective measures that can assist clinical decision-making when symptom reports may be untrustworthy.

Many widely used tools to assist clinicians in the assessment of SR-mTBI require or recommend pre-season baseline testing to use as a reference for subsequent evaluations [16]. Comprehensive baseline testing may be possible at the elite and professional levels of sport, but logistical constraints limit the feasibility of this approach for most athletes engaged in the recreational or amateur sporting environment. Recent evidence questions the clinical utility of commonly implemented SR-mTBI assessment tools even if a baseline reference is available due to sub-optimal test-retest reliability [16]. Objective measurement techniques including advanced functional imaging, transcranial magnetic stimulation, and electroencephalography have identified abnormalities in functional connsssectivity post-mTBI [17–29] which appear to underlie a wide array of symptoms observed clinically due to altered neurotransmission and information processing [30]. Whilst valuable insights are gained utilising advanced techniques, logistical constraints such as accessibility, cost, ease of use, and time to administer again limit the likelihood of their widespread integration into the clinical management of mTBI. To advance current best-practice mTBI management, the identification of objective, affordable, quick, and easy to administer neurophysiological tools demonstrating discriminative or predictive capacity without the need for a baseline reference is required.

**Fig. 1:**
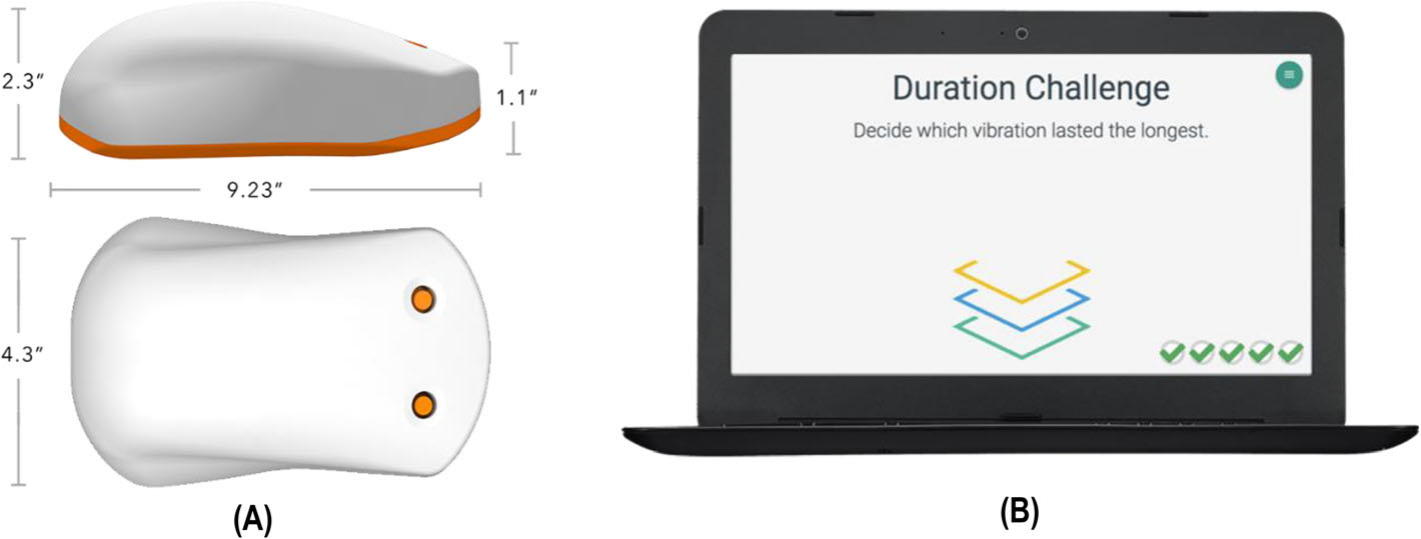
The ‘Brain Gauge’ **A** two-digit vibro-tactile stimulation handheld device (Brain Gauge. Cortical Metrics, Chapel Hill, NC, USA www.corticalmetrics.com) and **B** example of the visual cueing test screen

Sensorimotor processing represents one domain of functional connectivity that can be impaired following mTBI. Tactile somatosensory assessments (SA) take advantage of the highly organised structure of the somatosensory cortex providing one means of evaluating sensorimotor processing in individuals with neurological conditions, including those with mTBI [25, 26, 31–36]. The computer mouse-shaped Cortical Metrics Brain Gauge is a portable, quick, and easy to administer tool able to evaluate tactile somatosensory function. Mechanoreceptors in the fingertips of the non-dominant hand detect light vibrations delivered by the Brain Gauge, transmitting resultant sensory information to corresponding areas of the contra-lateral somatosensory cortex (Fig. 1). Here the sensory stimulus is processed before being relayed through commissures, such as the corpus callosum, to the motor cortex in the opposite hemisphere. A motor response is coordinated in the form of using the dominant hand to indicate an answer to a question about the stimulus [37]. Studies have indicated that aspects of the Brain Gauge SA (BG-SA) protocol can detect differences between individuals diagnosed with mTBI when compared to non-injured controls [26, 32, 34]. However, to date, no studies have evaluated the feasibility and utility of SA via the Brain Gauge to provide clinicians with objective information that might assist diagnostic and management decisions for SR-mTBI patients.

### Study Purpose and Research Questions

The purpose of this investigation was to evaluate the clinical utility of tactile BG-SA to assist clinicians when making SR-mTBI diagnosis and management decisions. Specifically, this study aimed to address four questions:

1. Can performance on tactile BG-SA during initial clinical assessment accurately discriminate between patients with SR-mTBI and a healthy reference sample;
2. Can performance on tactile BG-SA during initial clinical assessment alone, or in combination with symptom burden accurately classify recovery trajectory;
3. Do SR-mTBI patients perform differently on BG-SA at initial clinical assessment and/or clinical recovery compared to a healthy reference sample; and
4. Does BG-SA performance in SR-mTBI patients change between initial clinical assessment (symptomatic) and clinical discharge (asymptomatic)?

## Methods

### Research Design

A prospective cohort design enabled evaluation of the clinical utility of BG-SA to provide objective information to assist clinicians when diagnosing SR-mTBI, predicting recovery outcomes based on initial assessment performance, and determining if neurophysiological recovery coincides with clinical recovery. Data collection took place at a single dedicated SR-mTBI clinic between April 2019 and July 2019.

### Participants

A total of 79 consenting patients diagnosed with SR-mTBI by a sport and exercise medicine physician during their initial clinical assessment were recruited for the study. The sample was inclusive for age and sex. BG-SA data collected during the initial clinical assessment was available for all 79 participants. Follow-up BG-SA data were collected from 45/79 (57%) participants once they met clinical recovery criteria. Follow-up data were not available for the remaining participants because they were referred to a different service (4/79), lost to follow-up (12/79), or declined to complete their second BG-SA (18/79).

### Procedures

#### Ethical Conduct

Institutional (AUTEC 18/374) and health and disability committee (HDEC 18/NTA/108) ethical approvals were obtained, and this study was conducted according to the ethical standards of the Declaration of Helsinki. Participants provided written consent (participant assent and parental consent were acquired for participants < 16 years old) to having their data used for research and publication. All participant data were de-identified prior to extraction/data analysis to ensure confidentiality.

#### Clinical Management and Definitions of Recovery

Patients received usual clinical care as per a previously described service protocol which is briefly summarised in this paper. Additional details about the criteria/assessments used to identify SR-mTBI and treatment participants received have been published [38, 39].

As part of routine care, participants completed the Sports Concussion Assessment Tool (SCAT-5) symptom scale at the beginning of each visit, followed by a consultation with the supervising physician. During the initial clinical assessment, the physician completed a thorough history and physical examination. Participants received treatment in line with international recommendations in the form of education, written guidance, and individualised management to target the underlying causes of their signs and symptoms. Participants were scheduled for follow-up assessments every 7–14 days to evaluate their progress and, if necessary, to modify their treatment plan.

Before departing the initial assessment, participants completed tactile BG-SA requiring ~ 10 min. To control for environmental noise during BG-SA, participants underwent testing in a private area of the clinic free from visual distractions whilst wearing noise-cancelling headphones. Once participants achieved all clinical recovery criteria, they began a graduated return-to-play protocol and, before departing the clinic, completed the BG-SA battery for a second time.

Participants in this study were deemed to be clinically recovered once they achieved recovery criteria: (1) asymptomatic (defined as SSS ≤ 5 for males and ≤ 6 for females [8]), (2) demonstrated exercise tolerance, and (3) any abnormalities identified during the initial physical examination had resolved [38]. For this study, neurophysiological recovery was defined as participants demonstrating BG-SA performance similar to that reported in studies evaluating BG-SA performance in non-injured healthy controls.

### Instrumentation

#### SCAT-5 Symptom Burden

Participants completed the SCAT-5 [40] symptom scale during all appointments to quantify subjective SR-mTBI symptom reports. Participants were queried about 22 symptoms commonly related to SR-mTBI and asked to rank each symptom on a Likert scale from 0 (no symptom) to 6 (severe symptom). A Positive Symptom Total (PST; number of symptoms reported out of 22) and a Symptom Severity Score (SSS; sum of severity reported across the 22 symptoms out of 132) were composite scores derived from the symptom scale to represent the SCAT-5 symptom burden.

#### Brain Gauge Somatosensory Assessment

The Cortical Metrics BG-SA protocol [25, 26, 32–34, 41] was adapted to reduce administration time from the typical ~ 20–25 min to ~ 10 min to preserve the clinical flow. Participants placed their non-dominant hand on the BG with the tips of D2 (index finger) and D3 (middle finger) aligned with two 5-mm probes that delivered a vibrotactile stimulus. A 2-alternative forced choice testing paradigm was employed wherein participants responded to the stimulus, via a computer interface, with a mouse in their dominant hand by clicking on whether the left (D3) or right (D2) stimulus came first or lasted longer.

Somatosensory processing was quantified utilising three vibrotactile tasks: (1) temporal order judgement (TOJ), (2) temporal order judgement in the presence of a confounding stimulus (TOJc), and (3) duration discrimination (DUR). To assess TOJ, two sequential vibrotactile pulses were delivered randomly to D2 and D3 and the participant was queried as to which digit was stimulated first. TOJc employed the same testing procedure as TOJ with the addition of a 25-Hz concurrent stimulus that lasted the length of each trial. Previous research [31, 34, 36] has indicated that the concurrent stimulus during TOJc leads to worse performance than TOJ in healthy individuals, whereas this performance drop is not observed in samples of individuals known to have a neurological condition. DUR was assessed by delivering sequential stimulus of different durations randomly to D2 and D3 and the participant had to discriminate which of the two stimuli lasted longer. Before each test began, participants had to correctly respond to three training trials (with feedback) to ensure they understood the testing protocol. Then twenty trials, without feedback, were completed for each testing component. If the participant responded correctly to a given TOJ or TOJc test trial, the inter-stimulus interval of the following trial was reduced to increase difficulty. Conversely, the inter-stimulus interval was increased if the participant responded incorrectly to make the following TOJ or TOJc test trial easier. The duration of the test stimulus was decreased following correct responses to DUR test trials and increased after incorrect responses. Somatosensory processing for each task was measured in milliseconds (ms) and is presented as the average of the three best trials to which the participant responded correctly.

### Healthy Reference Sample

To understand how a patient performs on a given outcome measure, healthcare practitioners commonly consult literature describing how healthy samples perform on the same measure. Therefore, for research questions 1 and 3, we implemented a novel approach where comparisons for the patient cohort were made against simulated healthy distributions generated using pooled BG-SA data (TOJ, TOJc, and DUR performance) for healthy participants extracted from studies [26, 31, 34, 42] (see Table 1).

**Table 1:** BG-SA and SCAT-5 symptom burden data pooled to create healthy reference samples

Study	Participants	Age (years)	TOJ	TOJc	DUR	SSS
**Lovell et al.[**43**]**	Healthy young women *n* = 355	~ 13–24^◊^	–	–	–	4 [0–78]^b^
	Healthy young men *n* = 1391					2 [0–56]^b^
**Nguyen et al.[**31**]**	Healthy adults *n* = 19	46.4 ± 2.4^a,#^	25.1 ± 13.9 ms^a,†^	84.7 ± 58.4 ms^a,†^	65.0 ± 33.1 ms^a,†□^	
**Iverson et al.[**11**]**	Female high school athletes *n* = 14,668	15.5 ± 1.3^a^	–	–	–	3 [0;9]^c^
	Male high school athletes *n* = 17,290					1 [0;6]^c^
**Jones et al.[**42**]**	Healthy adults *n* = 16 (6 females)	23.0 ± 5.2^a^	48.8 ± 15.2 ms^a†^	–	–	–
**Tommerdahl et al.[**34**]**	Healthy college athletes *n* = 58	20.1 ± 1.2^a,^*	36.4 ± 21.3 ms^a,†^	95.2 ± 32.7 ms^a,†^	64.6 ± 28.2 ms^a,†^	–
**Pearce et al.[**26**]**	Healthy adults *n* = 20 (4 females)	37.7 ± 8.0^a^	23.8 ± 10.1 ms^a,‡^	–	48.7 ± 19.1 ms^a,‡^	–
**Radoi et al.[**10**]**	Healthy community volunteers *n* = 60 (22 females)	36.2 ± 13.9^a^	–	–	–	4 [0–31]^b^
*Healthy reference pooled sample size*			*n* = 113	*n* = 77	*n* = 101	*n* = 33,764
*Healthy reference pooled results*			34.0 ± 17.0 ms^a^	92.6 ± 40.4 ms^a^	61.5 ± 27.9 ms^a^	3 (2–4)^d^
*Healthy reference adjusted pooled results*			31.3 [21.9; 43.3]^c^	87.4 [65.1; 115.0]^c^	56.9 [40.9; 75.8]^c^	–

Notes: ^a^Mean ± SD; ^b^median [min-max]; ^c^median [IQR]; ^d^median (95% CI); ^◊^age inferred as not explicitly reported; ^#^controls were age and gender matched to patients with migraine in this study, age details for controls were not reported; *age was reported for *n* = 89 participants, data were presented for *n* = 58; ^□^*n* = 23 for this measure; ^‡^study reported confidence intervals which were transformed into standard deviation; ^†^study reported standard error which was transformed into standard deviation

The means and standard errors of somatosensory performance for healthy participants were presented in three studies [31, 34, 42]. A fourth study [26] presented healthy data as means and 95% confidence intervals. This information was extracted from each article, then standard errors and confidence intervals were subsequently transformed into standard deviations (see Table 1). The resultant standard deviations and accompanying means were pooled to provide an estimate of how healthy individuals perform on the BG-SA TOJ, TOJc, and DUR tasks based on previous research. Transformation of standard errors/confidence intervals to standard deviations and pooling of results followed the methods described in the 2011 Cochrane Handbook for Systematic Reviews of Interventions [44].

The reporting of means in previous studies suggests that performance on BG-SA is normally distributed in healthy individuals. However, previous reports highlight that measures of sensorimotor function (i.e., reaction/response times) were commonly positively skewed [45–47]. A previous study comparing baseline BG-SA performance to that observed within 7 days of SR-mTBI presented histograms demonstrating positively skewed distributions both pre- and post-injury, although measures of central tendency were not reported [32]. Positive skew was also observed for the BG-SA data obtained from participants with SR-mTBI in our clinical cohort. Therefore, the distribution of BG-SA performance in healthy populations is likely positively skewed and only contains positive values.

Healthy reference samples for TOJ, TOJc, and DUR were randomly simulated based on a gamma distribution with shape (mean^2^/standard deviation^2^) and rate 1/(standard deviation^2^/mean) parameters based on the pooled means and standard deviations from previous studies (see Table 1). Gamma distributions are characterised by positive skew and values greater than zero. Simulation of healthy reference samples for each BG-SA variable was accomplished using the ‘*rgamma*’ function in the ‘stats’ R package. Sizes of the healthy reference samples were dependent on the research question being evaluated. For research question 1, a random gamma distribution was generated for TOJ, TOJc, and DUR with a sample size of 79 to simulate a healthy comparison group for each variable with the same number of observations as those collected from SR-mTBI patients at the initial assessment. Performance on TOJ, TOJc, and DUR was acquired at initial assessment as well as clinical discharge from 45 participants who sustained SR-mTBI, so the sample size of random gamma distributions generated for research question 3 was also 45. Since the central tendency and dispersion of skewed distributions are more appropriately described using medians and interquartile ranges, the procedure described above was used to simulate 100 samples of 45 observations for TOJ, TOJc, and DUR, respectively, to approximate the pooled median and average interquartile range for each variable. These adjusted pooled values are presented in Table 1.

Whilst the purpose of this study was to evaluate the clinical utility of BG-SA, clinical best practice utilises subjective symptom reports as one indicator to determine when a SR-mTBI patient has achieved clinical recovery [5, 6]. However, at clinical recovery, a SR-mTBI patient may not be completely symptom free (PST = 0, SSS = 0), due to the non-specific nature of mTBI-like symptoms. This has been shown in several studies investigating mTBI-like symptom reports in healthy individuals [8–11]. Data from these studies were also pooled as a reference (see Table 1). For consistency, symptom endorsement data were only pooled from studies reporting medians and interquartile/minimum-maximum ranges [48] because symptom reports also appear to be positively skewed in healthy individuals, and once an SR-mTBI patient achieves clinical recovery [10, 11, 39, 43].

### Data Analyses

#### Data Distribution and Non-parametric Statistical Techniques

Exploratory data analysis revealed skewed distributions containing outliers for both clinical (age, days until initial assessment, days until asymptomatic, PST, and SSS) and somatosensory variables (TOJ, TOJc, and DUR; Shapiro-Wilks *p* ≤ 0.001 for each variable). Since the purpose of this investigation was to determine the clinical utility of BG-SA in a real-world clinical environment, outliers were not removed from the data, as these cases are representative of SR-mTBI patients that would present clinically. Due to the shape of the distributions, as well as heterogeneity of variances/covariances between sub-groups, non-parametric statistical techniques were implemented for each research question. Kruskal-Wallis and Mann-Whitney U tests were used to assess between-group differences for continuous variables.

#### Logistic Regressions

Logistic regressions enabled evaluation of TOJ, TOJc, or DUR accuracy to discriminate between SR-mTBI versus healthy reference samples for each variable. A single random gamma distribution was generated for TOJ, TOJc, and DUR to serve as a healthy reference sample (*n* = 79 to match the full SR-mTBI sample size). Due to a modest sample size, leave-one-out-cross-validation (LOOCV) was undertaken to evaluate the model on n-1 participants and to test the model’s capacity to correctly identify the class of the left-out case. This process was repeated for each participant, and accuracy metrics including sensitivity, specificity, positive predictive value (PPV), negative predictive value (NPV), percent correctly classified, area under the curve (AUC), and receiver operating characteristic (ROC) were derived for each somatosensory test. This approach is representative of how well a model trained on the current data would perform at classifying a new patient presenting with an SR-mTBI. Training and cross-validation of logistic regressions were performed using the ‘caret’ and ‘pROC’ R packages.

#### Decision Trees

Recursive partitioning in the form of a classification tree was utilised to explore if TOJ, TOJc, DUR, and/or intra-subject difference between TOJ and TOJc alone, or in combination with PST and/or SSS, at initial assessment were indicators of participant recovery trajectory. A classification tree approach does not rely on the assumptions of normality and homogeneity of covariance, allows for classification into > 2 groups, and presents a statistical representation of how medical professionals rule in/out factors that might identify the cause of a condition [49]. Classification trees were trained and tested using ‘rpart’ in the R package to determine which variables could correctly classify participants who became asymptomatic in > 14 days and ≤14 days or were lost to follow-up/referral. In some cases, participants became asymptomatic in ≤14 days but, due to scheduling limitations, were not seen for a follow-up visit until beyond 14 days post-injury. In this case, if the participant self-reported becoming asymptomatic at day 11 post-injury, then 11 days until asymptomatic was recorded.

LOOCV was used to tune and evaluate the classification trees. Tree pruning took place during cross-validation to reduce the likelihood of overfitting to the training data. Ten complexity parameters were evaluated as part of cross-validation to penalise the tree if an additional partition or inclusion of another variable did not enhance the model predictive accuracy as measured by cross-validated error. The final decision tree was determined by the complexity parameter that produced the simplest model (least number of partitions) that demonstrated the greatest classification accuracy. The sensitivity, specificity, balanced accuracy, and overall accuracy were calculated based on the predictions made by the final model for each iteration of LOOCV.

#### Between- and Within-Group Comparisons

Mann-Whitney U tests were used to compare if differences in somatosensory performance existed between SR-mTBI and healthy reference samples (see Table 1). The points of reference were when symptomatic at initial assessment and/or when clinically recovered at discharge. A random sample was simulated for each comparison using the process described in the “Healthy Reference Sample” section and a Mann-Whitney U test between the reference samples and SR-mTBI data was performed. To reduce the risk of type 1 error that could occur because of the distribution of a single random sample, this process was repeated 100 times and the distributions of the 100-resultant *p*-values are visualised using boxplots for TOJ, TOJc, and DUR at each variable and timepoint. The percentage of comparisons that yielded a *p-*value < 0.05 are reported to indicate the likelihood that observed differences in BG-SA performance between SR-mTBI participants and the healthy reference samples are due to chance.

Changes in somatosensory performance and symptom endorsement for the 45 participants when symptomatic at initial assessment, and clinically recovered at discharge, were tested using the Wilcoxon signed-rank test. Due to variability of clinical data, a priori α was set to 0.05 for comparisons. Statistical analyses and visualisations were performed using Python v3.6.10 and RStudio v1.1.456.

## Results

Descriptive statistics grouped by recovery trajectory are summarised in Table 2 (the top panel shows data for the 79 participants who completed the BG-SA at initial assessment; the bottom panel shows data for the 45 participants tested at both initial and discharge). Distributions of continuous variables were highly skewed; therefore, descriptive results are presented as median [IQR].

**Table 2:** Descriptive statistics grouped by recovery trajectory for SR-mTBI patients assessed using BG-SA at initial clinical assessment (*n* = 79) and at initial and discharge assessments (*n* = 45)

**Descriptive statistics for patients assessed with BG-SA at initial SR-mTBI assessment only**				
	Total (*n* = 79)	Resolution ≤14 days (*n* = 22)	Resolution > 14 days (*n* = 41)	Lost to follow-up (*n* = 16)
**Sex**^a^				
Male	60 (76%)	21.0 (95%)	28 (68%)	11.0 (69%)
Female	19 (24%)	1.0 (5%)	13 (32%)	5.0 (31%)
**Sport**^a^				
Rugby codes	54 (68%)	20 (91%)	26 (63%)	8 (50%)
Others	15 (19%)	1 (5%)	10 (24%)	4 (25%)
Football	10 (13%)	1 (5%)	5 (12%)	4 (25%)
Age^b^	19.0 [16.0; 23.0]	19.0 [16.0; 23.0]	19.0 [15.0; 23.0]	20.5 [16.8; 30.8]
Days until initial assessment^b^	10.0 [5.5; 14.5]	6.5 [4.0; 10.0]*	11.0 [6.0; 17.0]	11.0 [6.0; 14.0]
Days until asymptomatic^b^	20.0 [12.0; 34.5]	10.5 [6.0; 12.0]^◊^	30.0 [20.0; 45.0]	–
Initial PST^b^	11.0 [6.0; 17.0]	6.0 [3.25; 7.0]*^,†^	14.0 [7.0; 17.0]^‡^	16.0 [10.5; 18.0]
Initial symptom severity^b^	20.0 [8.5; 42.0]	8.0 [5.0; 14.3]*^,†^	25.0 [13.0; 47.0]^‡^	36.0 [23.5; 48.3]
TOJ performance (ms)^b^	29.0 [20.3; 40.2]	26.5 [17.1; 32.9]	29.0 [20.5; 40.6]	32.7 [22.5; 53.8]
TOJc performance (ms)^b^	59.9 [34.1; 84.4]	57.8 [40.7; 77.5]	63.9 [32.0; 86.2]	54.9 [42.2; 84.1]
DUR performance (ms)^b^	50.0 [29.2; 66.7]	50.0 [41.7; 72.9]	50.0 [25.0; 66.7]	45.8 [25.0; 72.9]
**Descriptive statistics for participants assessed with BG-SA at initial and discharge SR-mTBI assessments**				
	Total (*n* = 45)	Resolution ≤14 days (*n* = 15)	Resolution > 14 days (*n* = 30)	
**Sex**^a^				
Male	35 (78%)	14.0 (93%)	21 (70%)	
Female	10 (22%)	1.0 (7%)	9 (30%)	
**Sport**^a^				
Rugby codes	33 (73%)	13 (87%)	20 (66%)	
Others	8 (18%)	1 (7%)	7 (23%)	
Football	4 (9%)	1 (7%)	3 (10%)	
Age^b^	19.0 [16.0; 23.0]	19.0 [15.5; 23.0]	19.5 [16.0; 24.0]	
Days until initial assessment^b^	10.0 [5.0; 12.0]	10.0 [4.0; 10.0]^◊^	11.0 [6.0; 16.5]	
Days until asymptomatic^b^	20.0 [12.0; 32.0]	10.0 [5.0; 12.0]^◊^	27.5 [20.0; 42.5]	
Initial PST^b^	7.0 [5.0; 14.0]	5.0 [2.5; 7.0]^◊^	11.0 [6.25; 17.0]	
Discharge PST^b^	1.0 [0.0; 2.0]	0.0 [0.0; 1.0]^◊^	1.0 [0.25; 3.0]	
Initial symptom severity^b^	15.0 [7.0; 29.0]	7.0 [3.0; 11.0]^◊^	22.0 [10.5; 44.5]	
Discharge symptom severity^b^	1.0 [0.0; 2.0]	0.0 [0.0; 1.0]^◊^	1.0 [0.25; 4.0]	
TOJ performance (ms)^b^	27.2 [18.2; 36.8]	24.7 [15.7; 31.9]^◊^	28.3 [20.2; 40.4]	
TOJc performance (ms)^b^	55.6 [34.9; 82.5]	51.3 [42.3; 60.5]^◊^	66.1 [32.7; 85.3]	
DUR performance (ms)^b^	50.0 [25.0; 66.7]	41.7 [29.2; 54.2]^◊^	50.0 [29.2; 66.7]	

Notes: ^a^Frequency (%), ^b^median [25th percentile; 75th percentile], *Dunn’s post hoc comparison of Kruskal-Wallis tests *p* < 0.05 ≤ 14-day vs > 14-day resolution, ^†^Dunn’s post hoc comparison of Kruskal-Wallis tests *p* < 0.05 ≤ 14 days vs lost to follow-up, ^‡^Dunn’s post hoc comparison of Kruskal-Wallis tests > 14-day resolution vs lost to follow-up, ^◊^Mann-Whitney U *p* < 0.05 for ≤14-day vs > 14-day resolution

### Demographic and Clinical Information

Females accounted for 24% of the total sample (*n* = 79) and 50% of participants were between 16 and 23 years of age. Playing rugby union or rugby league was the inciting factor of SR-mTBI for 68% of the participants.

Participants with > 14-day symptom resolution (11.0 [6.0, 17.0]) and those who were lost to referral/follow-up (11.0 [6.0, 14.0]) took longer to present for their initial assessment than those with ≤14-day resolution (6.5 [4.0, 10.0]). Participants lost to referral/follow-up reported the highest level of symptom burden at initial assessment (PST 16.0 [10.5, 18.0]; SSS 36.0 [23.5, 48.3]) followed by > 14-day resolution (PST 14.0 [7.0, 17.0]; SSS 25.0 [13.0, 47.0]) and ≤ 14-day resolution (PST 6.0 [3.3, 7.0]; SSS 8.0 [5.0, 14.3]). Participants who experienced > 14-day resolution required a median 30.0 [20.0, 45.0] days to become asymptomatic compared 10.5 [6.0, 12.0] for those in the ≤14-day group.

Demographic and clinical data for the 45 participants with data from both initial assessment and discharge followed the same trends as described above and are presented in Table 2.

### Discriminative Utility of Somatosensory Assessments

**Fig. 2:**
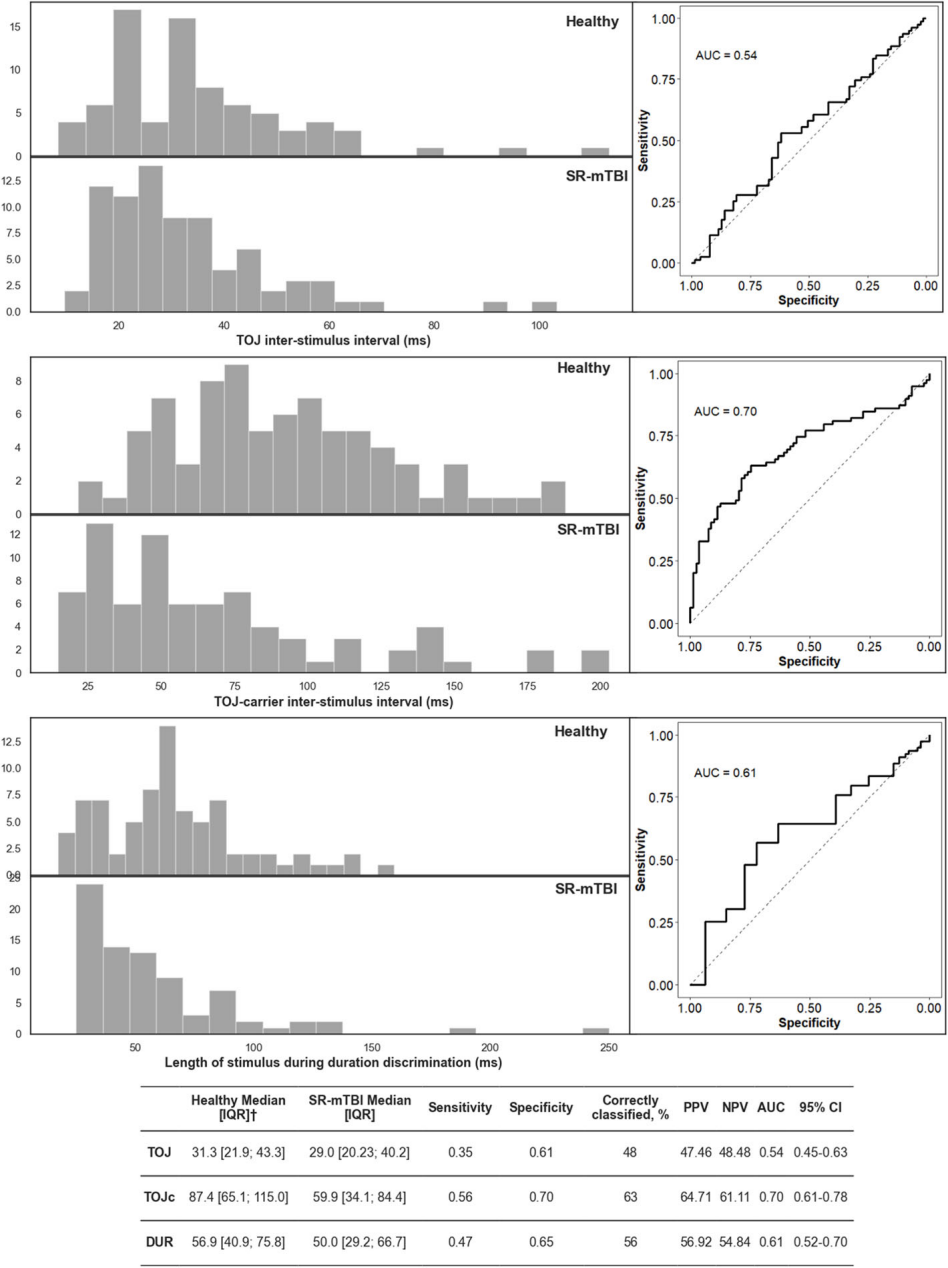
Discriminative performance of TOJ, TOJc, and DUR. Note: ^†^Median IQR for the healthy reference group in this figure is from a single randomly simulated skewed distribution based on the process described in the “Healthy Reference Sample” section of the “Methods” section. PPV positive predictive value, NPV negative predictive value, AUC area under the curve

Univariable logistic regression and ROC analysis were performed using TOJ, TOJc, and DUR, respectively, to evaluate how accurately any one of these BG-SA can discriminate participants with SR-mTBI when compared with a healthy reference sample simulated based on published results (see Table 1). TOJc demonstrated the best discriminative performance by correctly classifying 63% of cases to the correct group with an AUC of 0.70, 64.71 PPV, and 61.11 NPV (see Fig. 2). No discriminative capacity was observed for TOJ (48%, 0.54 AUC, 47.46 PPV, 48.48 NPV) and DUR (56%, 0.61 AUC, 56.92 PPV, 54.84 NPV).

### Prognostic Utility of Somatosensory Assessments

Two classification trees were developed using LOOCV to determine the prognostic capacity of BG-SA alone or in combination with symptom reports to correctly assign > 14-day resolution, ≤14-day resolution, or lost to referral/follow-up recovery trajectory membership to the 79 SR-mTBI participants (see Table 3). The first decision tree was developed using BG-SA alone. The final somatosensory decision tree used TOJ ≥ 50 ms, TOJc ≥ 68 ms, and TOJc < 47 ms as decision thresholds and demonstrated 48%, 54%, and 45% balanced accuracy results for > 14-day resolution, ≤14-day resolution, or lost to referral/follow-up, respectively. The second tree was developed using BG-SA and symptom reports to see if greater three group classification accuracy could be obtained (see Table 3). The final combination decision tree used SSS ≥ 20 as the lone decision threshold resulting in better balanced accuracy for classifying > 14-day resolution (63%), ≤14-day resolution (79%), or lost to referral/follow-up (50%) recovery trajectory membership.

**Table 3:** Prognostic utility of BG-SA to classify patient recovery trajectory (*n* = 79)

	Variables included and decision thresholds	Sensitivity	Specificity	Balanced accuracy	Overall accuracy	95% CI
**Three group classification using Brain Gauge performance only**						
> 14-day resolution	TOJ ≥ 50 ms TOJc ≥ 68 ms TOJc < 47 ms	0.83	0.13	48%	47%	36–58%
≤14-day resolution		0.14	0.95	54%		
Lost to follow-up		0	1	45%		
**Three group classification using Brain Gauge performance combined with clinical variables**						
> 14-day resolution	SSS ≥ 20	0.68	0.58	63%	60%	48–70%
≤14-day resolution		0.86	0.72	79%		
Lost to follow-up		0	1	50%		

### SR-mTBI Somatosensory Performance at Initial Assessment and Discharge

**Fig. 3:**
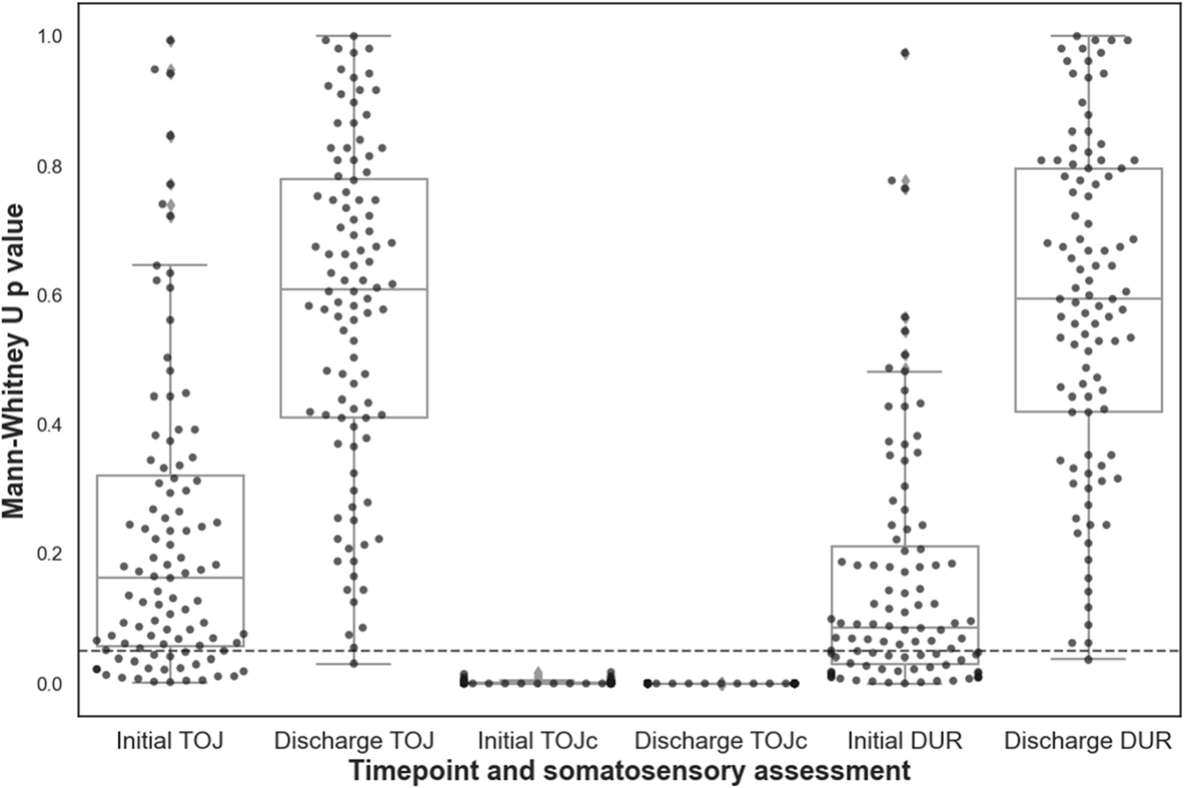
Results of comparing SR-mTBI TOJ, TOJc, and DUR performance against 100 simulated healthy reference samples. Datapoints represent the resultant *p*-values of each experiment. The dashed line represents *p* = 0.05

Between-group differences in TOJc performance (consistent with *p* < 0.05) were observed in 100% of the comparisons between SR-mTBI participants and simulated healthy reference samples both at initial assessment and discharge (see Fig. 3). Statistically significant differences in TOJ and DUR performance were observed in 23% and 37% of comparisons at initial assessment, respectively. At clinical discharge, 1% of TOJ and DUR comparisons between participants with SR-mTBI and healthy reference samples yielded a *p*-value < 0.05 (see Fig. 3).

### Change in Somatosensory Performance and Symptom Burden Across Timepoints

**Fig. 4:**
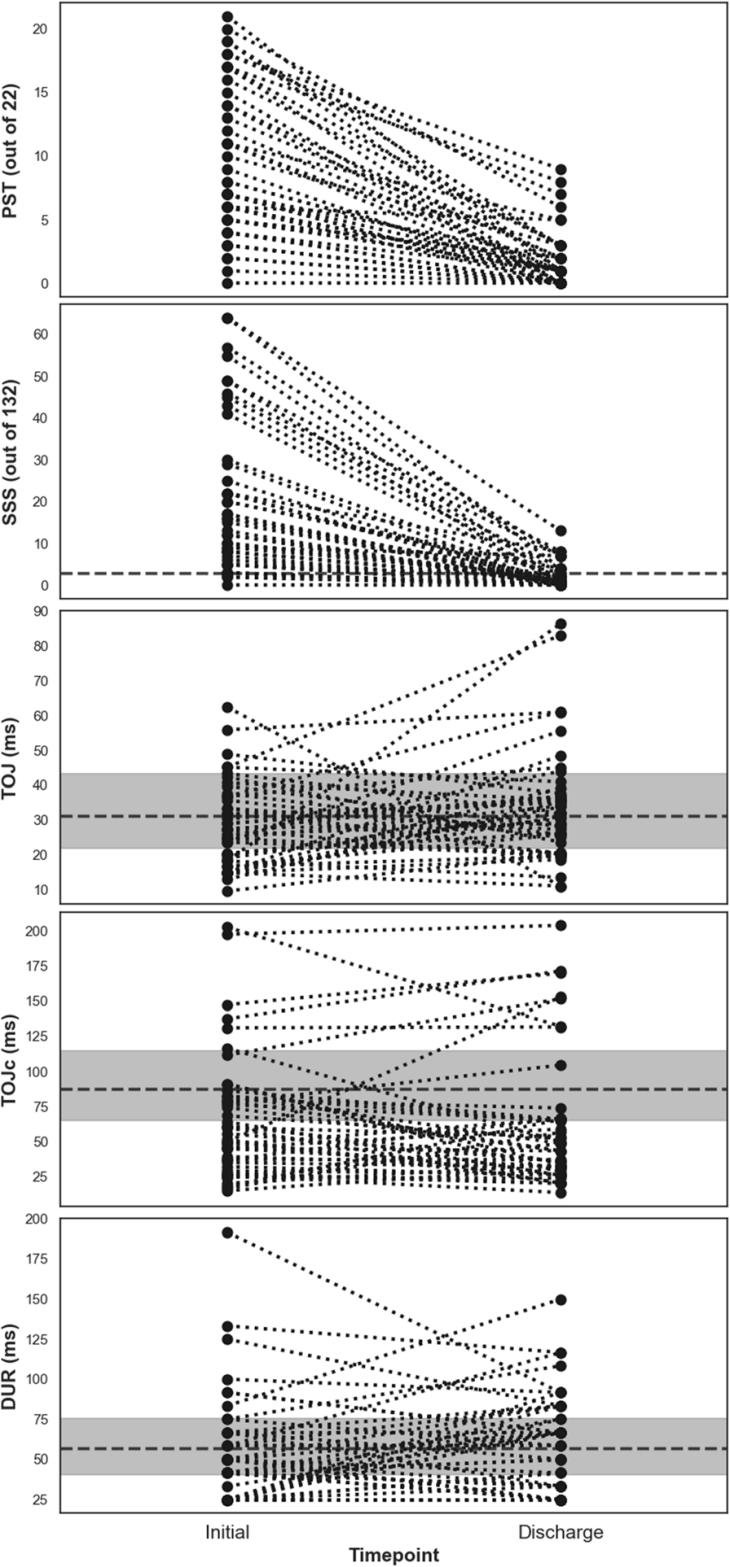
Initial assessment versus discharge BG-SA performance (TOJ, TOJc, DUR) and SCAT-5 symptom burden (SSS and PST). Note: Dashed black lines represent the pooled median performance in healthy individuals shown in Table 1. Grey bands represent the average IQR from 100 simulations of healthy distributions shown in Table 1

Figure 4 visualises changes in somatosensory performance and symptom endorsement from initial clinical assessment to discharge. There were no within-group changes in BG-SA between initial assessment and clinical discharge when participants were clinically recovered at discharge (TOJ *p* = 0.074, TOJc *p* = 0.170, DUR *p* = 0.234). A considerable drop was seen between timepoints for PST (*p* < 0.001) and SSS (*p* < 0.001).

## Discussion

The purpose of this study was to evaluate the clinical utility of BG-SA to provide objective information to assist clinicians when diagnosing SR-mTBI, to predict recovery outcomes based on initial assessment performance, and to determine if neurophysiological recovery coincides with clinical recovery. Whilst previous studies have demonstrated aspects of BG-SA can identify differences or discriminate between individuals with known neurological conditions and controls, none has evaluated the prognostic capability of BG-SA nor evaluated BG-SA performance once participants met clinical recovery criteria. To our knowledge, this is the first study to evaluate BG-SA in an ecologically valid setting and to independently assess BG-SA outside the group responsible for its development and commercialisation.

### Marginal Utility of Somatosensory Assessments to Assist Diagnostic Decisions

SR-mTBI diagnosis must be made by a physician based on a comprehensive clinical examination, but previous research suggests BG-SA may provide physicians with additional objective information to guide their evaluation and potentially assist when establishing SR-mTBI diagnosis. Such information would be particularly useful if there is delayed symptom onset or when non-compliant patients appear to be underreporting their symptomology [12–15]. Favorov et al. evaluated whether BG-SA could be used to discriminate between pre-season baseline performance versus performance within 1 week of SR-mTBI and reported that reaction time variability yielded the greatest accuracy (0.91 AUC), followed by amplitude discrimination (0.83 AUC), DUR (0.78 AUC), mean reaction time (0.69 AUC), and TOJ (0.53 AUC) [32]. Results for TOJc were not available in that study and BG-SA reaction time variability was not evaluated in the present study because of a lack of studies reporting how healthy controls perform on this measure, preventing pooling and simulation. Our findings suggest limited discriminative accuracy of the BG-SA TOJ, TOJc, and DUR to assist clinicians faced with the challenge of diagnosing SR-mTBI when patients present for initial assessment ~ 1–1.5 weeks after the suspected injury. Our results indicated that only TOJc demonstrated marginal discriminative accuracy of BG-SA assessed (0.70 AUC, 64.71 PPV, 61.11 NPV). The low to moderate PPVs and NPVs observed for TOJ, TOJc, and DUR suggest that these measures would offer little assistance to clinicians working under similar clinical conditions as those in this study when establishing SR-mTBI diagnosis.

Differences in outcomes between the current study and previous reports may be explained by the conditions under which BG-SA were performed. Participants in Favorov et al.’s study appeared to be assessed under highly controlled/laboratory conditions which permitted the collection of pre-injury baseline data, assessment of a greater number of BG-SA tasks, and earlier post-injury evaluation [32]. Our study was embedded within a busy SR-mTBI clinic which meant the number of BG-SA tasks had to be limited to preserve the clinical flow. Participants were members of the general public presenting with SR-mTBI from a variety of sports, levels of competition, and geographic areas meaning baseline testing was not possible and comparison to previously published data from healthy individuals was necessary. Participants underwent initial clinical assessment a median of 10 days post-injury because of the realities of clinical scheduling constraints. Whilst previous reports suggest promising potential for BG-SA to assist diagnostic decisions, there is a lack of ecological validity. For these reasons, our findings are likely more representative of how TOJ, TOJc, and DUR would perform at discriminating between healthy individuals and those who have suffered SR-mTBI under conditions which many clinicians operate. It is possible that a greatly reduced BG-SA protocol (< 5 min) may be useful in high volume environments where patients present on the day of injury such as emergency departments and walk-in clinics, but research into this potential application is needed.

### Limited Prognostic Utility of Somatosensory Assessments

Whilst a screening tool that can accurately discriminate between cases with or without a given condition is certainly useful, an optimal tool would pair discriminative and prognostic capabilities. In the case of SR-mTBI, early and accurate prediction of recovery trajectories would possibly reduce the need for patients likely to recover quickly to attend follow-up appointments, subsequently, keeping limited follow-up appointments available for the patients who require them most. Our analysis evaluated whether BG-SA performance at initial clinical assessment (regardless of days until initial assessment) could classify participants who would become asymptomatic in ≤14 days and > 14 days or those who were lost to follow-up/referral. Early identification of those lost to referral/follow-up would be particularly useful to identify patients requiring referral to a different service early on, or to begin to understand why some patients do not present for follow-up. Our data suggest the limited utility of TOJ, TOJc, and DUR to classify participants accurately into their respective resolution trajectory group. The lack of classification accuracy can likely be explained by the overlap in performance on the BG-SA tasks evaluated across the three groups at initial clinical assessment (Table 2).

### Neurophysiological Abnormalities Still Present at Clinical Discharge

A growing body of literature highlights that some individuals with a history of mTBI present with persistent neurophysiological abnormalities for weeks, months, and years post-injury when compared to healthy controls [22, 27, 50–53]. Several studies have reported that these abnormalities still exist once an mTBI patient becomes asymptomatic and/or meets clinical recovery criteria [54–59]. It appears that TOJ and DUR lack sensitivity to detect group-level performance differences between individuals who have recently suffered SR-mTBI and performance consistent with healthy individuals, as shown by the lack of difference in performance when participants were symptomatic at initial assessment (see Figs. 3 and 4). In contrast, TOJc performance was impaired both at initial assessment ~ 10 days post-injury and remained impaired at clinical discharge in individuals with SR-mTBI (see Figs. 3 and 4). Our findings support the notion that clinical recovery precedes complete neurophysiological recovery based on the dissimilarity of BG-SA TOJc performance between SR-mTBI participants at clinical discharge and healthy reference values.

The impact of these persistent neurophysiological abnormalities beyond clinical recovery remains to be understood. This phenomenon may be analogous to managing an athlete after a fracture. The athlete may report being pain free during clinical examination, whilst X-ray images may suggest the athlete requires more time for fracture union before returning to play. In this scenario, the objective information gained using X-ray may assist the clinician in refining an individualised management plan that reduces the risk of the athlete returning to play when they may be susceptible to reinjury. Future longitudinal cohort studies are needed to quantify BG-SA post-mTBI, through clinical recovery, and the weeks/months after clinical recovery to determine the utility of BG-SA as an objective means to track neurophysiological recovery in a clinically useful manner. Overall, reports of lingering neurophysiological disruption across multiple independent studies utilising a variety of objective outcome measures raise the question of whether definitions and expectations of recovery post-mTBI require further revision.

## Limitations

This study was embedded in a busy SR-mTBI clinic that required several compromises to preserve the clinical flow and ecological validity of study findings. These compromises led to limitations that should be accounted for when considering the findings of this investigation. A shortened version of the BG-SA protocol had to be implemented because the complete battery required too much time and burden on behalf of the participants. It is possible that the BG-SA components omitted from the current study may have yielded greater discriminative or prognostic accuracy than TOJ, TOJc, or DUR. In practice, SR-mTBI patients commonly do not present for clinical evaluation until days after sustaining the injury because of delays in seeking medical treatment and/or due to clinical scheduling constraints [60]. Collection of BG-SA data on the date of injury may have been more favourable to understand the immediate consequences of SR-mTBI and how performance changes between the onset of injury and initial clinical assessment. Collection of BG-SA data on the day of injury may potentially have enabled the development of a better prognostic model.

The current investigation only evaluated the clinical utility of BG-SA in relation to SCAT-5 symptom burden because resolution of symptoms was a main criterion used to determine clinical recovery. It is possible that performance on BG-SA may have related to specific symptoms or predicted outcomes for other aspects of the SCAT-5 such as cognitive and balance assessments. It is worth noting that the cognitive and balance assessments within the SCAT-5 lack clinical utility to detect meaningful differences at the time which most participants presented for initial clinical assessment in this study [39, 61, 62]. Nevertheless, more independent research into the reliability and validity of BG-SA under clinical conditions and their relation to clinical outcome measures is needed before integrating the use of BG-SA into clinical practice.

We adopted a novel approach to pool and simulate healthy reference data to replicate clinical decision-making conditions. Assumptions were made during the simulation of healthy reference samples used for research questions 1 and 3, particularly the skewness of healthy BG-SA performance and the use of gamma distributions during simulations. There may be issues with the representativeness of the pooled healthy reference data due to the relatively low sample size. A more traditional approach would have included the recruitment of a healthy control group but issues with representativeness can also be a common limitation of such designs due to matching issues. Since the BG-SA is a standardised and computerised testing protocol, we argue that pooling of healthy data from multiple independent studies may have provided a more representative comparison group than a convenience sample of healthy individuals. Given the preliminary nature of this investigation and the conservative findings presented, these assumptions and limitations seem justifiable. Our modest sample size included unbalanced resolution trajectory subgroups which may have prevented the development of an accurate prognostic model. Furthermore, due to sample size, it was not possible to perform further subgroup analysis based on age, sex, sport, or predominant symptom cluster [63]. Acquisition of BG-SA requires sustained attention and screen time exposure. Disruptions in attention during BG-SA and/or intolerance to screens due to SR-mTBI may have influenced testing.

## Conclusions

Our findings suggest that the discriminative and prognostic utility of BG-SA TOJ, TOJc, and DUR to assist diagnostic decision-making and to predict recovery trajectory under ecologically valid conditions appears limited. Abnormal BG-SA TOJc performance was observed when participants with SR-mTBI met clinical recovery criteria. This finding adds to a growing body of literature reporting that clinical recovery is not necessarily indicative of complete neurophysiological recovery. Given the realities of time and budget constraints in clinical practice, our findings do not provide sufficient justification to recommend the allocation of time and resources to acquire BG-SA TOJ, TOJc, or DUR to assist clinical management of SR-mTBI patients at this time. Replication of these preliminary findings and future work including BG-SA reaction time variability and/or amplitude discrimination would determine whether BG-SA is a tool that clinicians could integrate into regular practice.

## Data Availability

Data sharing is not applicable to this article as data analysed in the current study contains participant medical information. Sharing of this information would breach patient privacy and confidentiality and breach the ethics approval gained for the study.

## Abbreviations

AUC: Area under the curve
BG-SA: Brain gauge somatosensory assessment
DUR: Duration discrimination
LOOCV: Leave-one-out-cross-validation
NPV: Negative predictive value
PPV: Positive predictive value
PST: Positive symptom total
SCAT-5: Sport concussion assessment tool
SR-mTBI: Sport-related mild traumatic brain injury
SSS: Symptom severity score
TOJ: Temporal order judgement
TOJc: Temporal order judgement in the presence of a confounding stimulus
